# Chronic *Corynebacterium striatum* Septic Arthritis in a Patient Referred for Total Knee Arthroplasty

**DOI:** 10.1155/2020/1392182

**Published:** 2020-03-05

**Authors:** Katharine Hollnagel, Jacob Willen, Michael Ellis, Yalda Soleimanifard, Robert Booth, Sumon Nandi

**Affiliations:** ^1^Department of Orthopaedic Surgery, The University of Toledo Medical Center, USA; ^2^Department of Infectious Disease, The University of Toledo Medical Center, USA; ^3^Department of Pathology, The University of Toledo Medical Center, USA; ^4^Department of Orthopaedics, University of Maryland School of Medicine, USA

## Abstract

**Background:**

While *Corynebacterium striatum* and other *Corynebacterium* species were historically considered contaminants, they are recently being identified as pathogens with increasing frequency. *Case Summary*. We report the case of a 78-year-old gentleman with a three-year history of knee pain and established diagnosis of osteoarthritis referred for consideration for total knee arthroplasty. He had no knee pain with passive range-of-motion. Plain films demonstrated bony erosions atypical for osteoarthritis. Joint aspiration white blood cell count was 30,548/mm^3^, but multiple cultures were positive for *C. striatum*. The infection was successfully treated with open irrigation and debridement, complete synovectomy, and six weeks of intravenous daptomycin.

**Conclusion:**

To our knowledge, this is the first report of chronic *C. striatum* septic arthritis of a native joint and only the third case of *C. striatum* septic arthritis of the knee.*C. striatum*. The infection was successfully treated with open irrigation and debridement, complete synovectomy, and six weeks of intravenous daptomycin. *C. striatum*. The infection was successfully treated with open irrigation and debridement, complete synovectomy, and six weeks of intravenous daptomycin.

## 1. Introduction

Chronic septic arthritis is a rare condition characterized by insidious onset of joint pain and joint destruction. The most common pathogens associated with chronic septic arthritis are mycobacteria or fungi [1]. *Corynebacterium striatum* is a catalase-positive, gram-positive bacillus that is among the normal flora of the skin and mucous membranes [1]. While *C. striatum* and other *Corynebacterium* species were historically considered contaminants, they are recently being identified as pathogens with increasing frequency [2]. Specifically, multiple cases are reported in the literature of *C. striatum* causing opportunistic infections in hospitalized patients and those with indwelling or implanted devices [3–6]. To date, there have only been six reported cases of *C. striatum* causing acute septic arthritis in a native joint [7–12]. Herein, we describe the first case of chronic *C. striatum* septic arthritis and osteomyelitis of the knee in a patient referred for total knee arthroplasty.

## 2. Case Presentation

This is the case of a 78-year-old gentleman with a past medical history of osteoarthritis, diabetes mellitus, and prostate cancer who presented to our institution with three years of left knee pain.

The history begins in 2013, when the patient began to have progressive left generalized knee pain. Symptoms were exacerbated with activity and relieved with rest. The patient denied fevers as well as history of trauma or surgery to the knee. He had tried activity modification, physical therapy, and hinged knee brace without relief. Two months prior to presentation in our clinic, in June 2016, the patient had an intra-articular steroid injection within the left knee that provided relief for 2 days. He performed bed-to-chair transfers only with the use of a walker at the time of presentation in August 2016. The patient was retired, and the remainder of the family and social history were noncontributory. He was referred for consideration for total knee arthroplasty.

The patient's medications included aspirin 81 mg po daily, atorvastatin 40 mg po daily, carvedilol 12.5 mg po daily, furosemide 80 mg po daily, meloxicam 7.5 mg po daily, metformin 500 mg po bid, metolazone 5 mg po daily, mirtazapine 30 mg po daily, extended release oxycodone 10 mg po q12 hours, and phenytoin 125 mg po tid. He had no known drug allergies.

**Figure 1:**
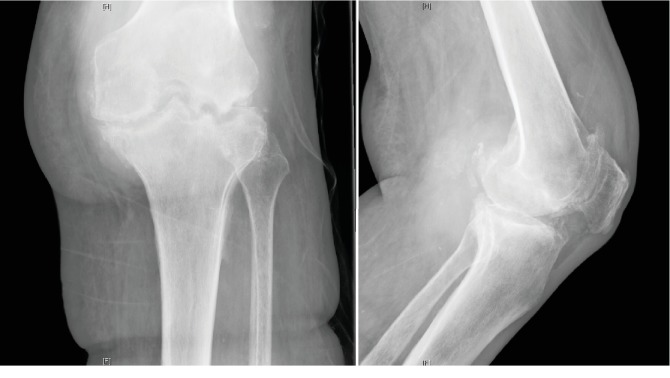
Left knee anteroposterior (AP) and lateral plain films demonstrating joint space narrowing with erosive features.

On physical examination, this Caucasian gentleman was 5 feet 11 inches tall and weighed 262 pounds. His left knee skin was intact and without erythema. The patient had left knee pain with weight bearing and transferred from his wheelchair to the examination table with an antalgic gait. Left knee medial and lateral joint lines and femoral condyles were tender to palpation. He had no pain with passive range of motion of the left knee, which demonstrated a 20-degree flexion contracture and 100 degrees of flexion. Plain films of the left knee demonstrated joint space narrowing with erosive features on both sides of the joint (Figure 1).

Given the atypical appearance of his radiographs, the patient elected to obtain inflammatory markers. Serum white blood cell count was 8,800/mm^3^ (reference range 4,000-10,000/mm^3^), erythrocyte sedimentation rate (ESR) was 68 mm/hr (reference range 0-10 mm/hr), and C-reactive protein (CRP) was 63.7 mg/L (reference range 0.0-7.0 mg/L). The patient's history of diabetes mellitus and prostate cancer, together with his elevated inflammatory markers and radiographic findings, suggested a differential diagnosis of septic arthritis versus malignancy. The patient was admitted to the hospital for further workup.

**Figure 2:**
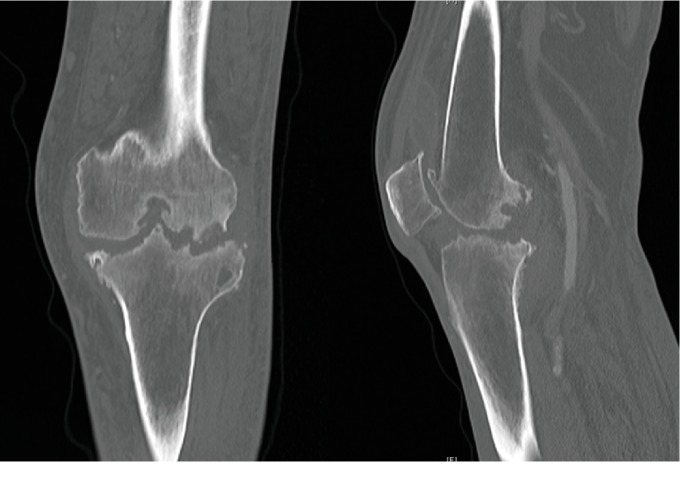
Left knee coronal and sagittal computed tomography (CT) images demonstrating bony destruction of the distal femur and proximal tibia.

Computed tomography (CT) scan of his left knee demonstrated bony destruction of both the distal femur and proximal tibia without evidence of malignancy (Figure 2). Two successive left knee joint aspiration cultures were positive for *Corynebacterium striatum* and negative for crystals. The higher of the two aspirate white blood cell (WBC) counts was 30,548/mm^3^ with 96% neutrophils.

## 3. Final Diagnosis

Our diagnosis was chronic *C. striatum* septic arthritis of the left knee.

## 4. Treatment

The patient elected to proceed with open left knee irrigation and debridement (I&D), which was performed through a medial parapatellar arthrotomy. Turbid synovial fluid and exuberant synovitis were observed. A total synovectomy was performed. Tibial and femoral articular surfaces were debrided of all necrotic or devitalized tissue and then contoured smoothly. A small drill was used to enter the femoral and tibial canals, and no purulence was encountered. Multiple specimens of soft tissue and bone were sent for culture and pathology. A hemovac drain was placed in the joint, and the wound closed in multiple layers with monofilament absorbable suture.

**Figure 3:**
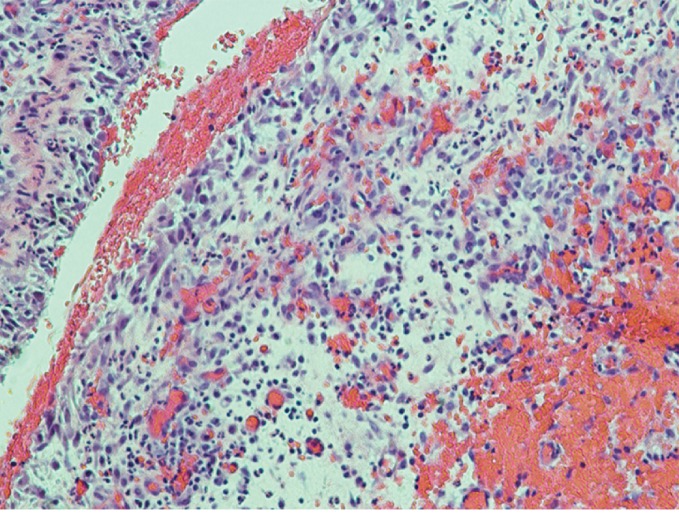
Histology of left knee joint intraoperative tissue specimen demonstrating acute and chronic inflammation consistent with infection.

Intraoperative cultures were again positive for *C. striatum* and negative for mycobacteria and fungi. Pathology demonstrated acute and chronic inflammation consistent with infection, without evidence of malignancy (Figure 3). The infectious disease service was consulted, and the patient was discharged on 500 mg of intravenous (IV) daptomycin daily for six weeks based on susceptibility testing.

## 5. Outcome and Follow-Up

**Figure 4:**
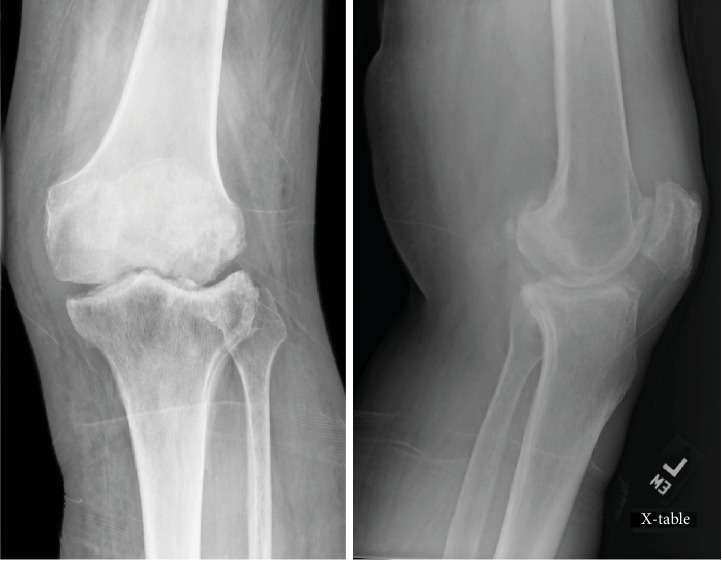
Left knee anteroposterior (AP) and lateral plain films status post open irrigation and debridement.

At most recent 1-year follow-up, the patient denied any left knee pain and was ambulating independently with the use of a cane. Active knee ROM was painless from zero to 115 degrees of flexion. Plain films demonstrated smoothly contoured femoral and tibial surfaces within the left knee joint (Figure 4).

## 6. Discussion

In the literature, there are six reported cases of *C. striatum* acute septic arthritis of native joints. Scholle and Westblade et al. both described cases in which patients developed acute knee pain and swelling after a fall [7, 8]. In both cases, initial knee aspiration was culture negative and *C. striatum* grew only upon repeat aspiration or from intraoperative cultures. Feced Olmos et al. and Molina Collada et al. describe cases of *C. striatum* acute septic arthritis following a corticosteroid injection of the shoulder joint and knee joint, respectively [9, 10]. Cone et al. described a patient who developed *C. striatum* acute septic arthritis of the elbow following a scalpel injury [11]. Finally, Roy and Ahmad describe a case of *C. striatum* acute septic arthritis of the shoulder in a lung transplant patient on hemodialysis [12]. In all cases, *C. striatum* was found to be multidrug resistant, and symptoms resolved with surgical debridement followed by antibiotic therapy. To our knowledge, this is the first report of chronic *C. striatum* septic arthritis of a native joint and only the fourth case of *C. striatum* septic arthritis of the knee.

*Corynebacterium* species, while previously considered culture contaminants, are increasingly being identified as causes of opportunistic infections. Long-term hospitalization, immunocompromise, or the presence of indwelling or implanted devices are all recognized risk factors of *Corynebacterium* infections [2]. In the five out of the six previously documented cases of acute *C. striatum* septic arthritis, common to all patients was instrumentation of, or trauma to, the affected joint. The only patient without such history was a transplant patient immunosuppressive agents receiving hemodialysis. The patient with chronic *C. striatum* septic arthritis described herein denied recent hospitalization or joint instrumentation/trauma; however, his history of diabetes mellitus may have contributed to an overall immunocompromised state.

Many features of this case provide guidance to the practicing clinician treating patients with an established diagnosis of osteoarthritis and chronic knee pain. First, referral for total knee arthroplasty should not preclude consideration of diagnoses other than osteoarthritis. Second, there should be a low threshold for initiating an infection workup in patients with radiographic features atypical for osteoarthritis, even in the absence of pain with short-arc range of motion. Recent intra-articular steroid injection should raise the suspicion for septic arthritis, as described herein and previously in the literature [9, 10]. The infection risk associated with native knee intra-articular steroid injection is underscored by the increased incidence of periprosthetic joint infection when total knee arthroplasty is performed up to 3 months following injection [13]. *C. striatum* septic arthritis is challenging to diagnose because it may present as a chronic, indolent infection with synovial WBC count less than 50,000/mm^3^. As a result, the importance of final aspiration culture results cannot be overemphasized. Multiple joint aspirations may be required to identify *C. striatum* and should be performed if there is a high suspicion for infection [7, 8].

## 7. Conclusion

We describe for the first time a case of chronic *C. striatum* septic arthritis of a native joint. The infection was successfully treated with open I&D, followed by a six-week course of IV daptomycin. Our report further supports the notion that *Corynebacterium* species may indeed be pathogenic and should not always be dismissed as culture contaminants. Atypical radiographic findings, especially erosive features within the joint, should prompt an infection workup even with a benign physical exam. It is important to note that a synovial WBC count less than 50,000/mm^3^ does not exclude chronic *C. striatum* septic arthritis. When there is a high suspicion for infection, as there is with recent intra-articular steroid injection, repeat joint aspirations are indicated if initial culture results are negative.

It is essential to identify new technologies, such as next generation sequencing (NGS), that aid the diagnosis of infection when there is ambiguity in clinical presentation. NGS sequences the entire genome of pathogens within a sample. This highly sensitive modality has proven effective in identifying the infecting organism in culture-negative infections [14]. However, further innovation is needed before NGS can be used to determine when a common contaminant, such as *Corynebacterium*, is the pathogen [15].
